# Rumination as a within-person indirect pathway between daily negative life events and suicidal ideation in recently hospitalized adults

**DOI:** 10.1016/j.jpsychires.2025.05.050

**Published:** 2025-05-23

**Authors:** Gemma T. Wallace, Melanie L. Bozzay, Leslie A. Brick, Ivan W. Miller, Emily Mower Provost, Heather T. Schatten

**Affiliations:** a Department of Psychiatry and Human Behavior, Alpert Medical School of Brown University, 700 Butler Drive, Providence, RI, 02906, United States; b The Ohio State University Wexner Medical Center, 1960 Kenny Rd., Columbus, OH, 43215, United States; c Psychosocial Research Program, Butler Hospital, 345 Blackstone Blvd, Providence, RI, 02906, United States; d Computer Science and Engineering, University of Michigan, 2260 Hayward St., Ann Arbor, MI, 48109, United States

**Keywords:** Suicidal ideation, Rumination, Stressful event, Psychiatric inpatient, Ecological momentary assessment, Post-discharge, Heterogeneity

## Abstract

Negative life events are often implicated as a near-term risk factor for suicidal ideation (SI); however ruminative processes may play a critical role in amplifying the distress following experiences of negative life events, ultimately leading to greater suicide risk. In the present work, we examined whether rumination indirectly impacted the association between negative events and SI intensity in a day-level ecological momentary assessment (EMA) study. Participants (*N* = 107; M_age_ = 35.9 years, 65% female, 81% white) completed an EMA protocol for 65 days following psychiatric hospitalization. We used dynamic structural equation modeling to test a day-level, within-persons model of direct and indirect effects between negative events, rumination, and SI intensity. After fitting the model across all people, we derived person-specific estimates to examine heterogeneity in the model parameters (i.e., while the indirect effect may be significant in the full sample, effect sizes may vary across individuals). Results indicated that the indirect effect of rumination was significant in the full sample (unstandardized estimate = 0.026) and represented 38.058% of the total effect. Moreover, results for the person-specific examination indicated that the indirect effect was present for nearly all participants who reported SI at any point in their EMA data. Thus, in addition to highlighting the critical role that rumination can play in SI, this study also highlights the value in conducting person-specific research to understand the complexity and heterogeneity of psychological processes involved in suicide risk.

## 1. Introduction

Suicide is a critical public health issue, resulting in the deaths of approximately 132 people per day in the United States (Garnett and Curtin, 2023). Although advances have been made in suicide treatment (D’Anci et al., 2019), preventing suicides remains a significant clinical challenge because there are not clear strategies for determining *which* patients are at greatest risk for suicide and *when* suicide risk will be highest. Studies have identified more intense suicidal ideation (SI) in individuals who recently attempted suicide (Kleiman et al., 2018), and history of ever experiencing high-intensity SI differentiated individuals who attempted suicide from those who only had SI (Law et al., 2018). Thus, the intensity of SI is an important indicator of suicide risk severity (Posner et al., 2011). However, intensity of SI can fluctuate considerably over brief periods (i.e., hours to days) (Kleiman et al., 2017; Rogers et al., 2024), limiting the time clinicians have to act during periods of elevated risk. Thus, research is needed to identify factors that proximally influence intensity of SI, and to understand how these factors vary across individuals, to better guide strategies to prevent suicide.

Robust research implicates negative life events in short-term risk for SI. Distress associated with negative life events has been described in multiple theories of suicide as a key causal process in suicide risk (Baumeister, 1990; Shneidman, 1993). A large systematic review found consistent links between negative life events and SI (Liu and Miller, 2014), and recent research suggests negative life events precede the onset or worsening of SI, even for periods as short as hours to days (Bagge et al., 2014; Cheek et al., 2020; Franz et al., 2021; Glenn et al., 2022; Oquendo et al., 2021; Yen et al., 2005). Nevertheless, negative life events are a universal human experience, and most individuals do not experience SI in their lifetimes (Czeisler et al., 2020). In fact, negative life events in general do not always result in SI, even among patients who recently attempted suicide (Husky et al., 2017), suggesting that negative life events alone are not sufficient to understand or anticipate increased suicide risk.

Other factors, such as an individual’s *response* to negative life events, may be needed to characterize the conditions that confer increased risk of SI. In particular, we posit that ruminative thought processes might amplify the distress associated with negative life events, thereby increasing the intensity of SI. Rumination is a form of repetitive negative thinking – a transdiagnostic style of thought that is excessive and difficult to control and focused primarily on negative content (Ehring and Watkins, 2008). When an individual ruminates, they fixate on the causes, meaning, and consequences of their distress in a manner that is unproductive (Nolen-Hoeksema and Morrow, 1991). Research further indicates that rumination reflects impairments in one’s ability to disengage attention from depressogenic information (Grafton et al., 2016), meaning the individual may have difficulty halting these maladaptive thought processes. Indeed, the emotional cascade model posits that ruminative thought processes amplify the experience of distress, and distress in turn increases rumination, forming a self-perpetuating cycle that exacerbates negative emotions (Selby and Joiner, 2013). Ultimately, this experience may be so aversive that individuals consider extricating themselves via suicide (Selby and Joiner, 2013).

There is some evidence to support this interpretation. Consistent with the emotional cascade model, rumination has been found to amplify and prolong negative emotions and physiological stress (Watkins and Roberts, 2020). More specifically, rumination appears to increase negative emotional responses to stressful events in patients with a range of psychiatric diagnoses (Baik and Newman, 2023; Connolly and Alloy, 2017; Ruscio et al., 2015). Rumination has also been concurrently and prospectively linked with SI, including in research examining these associations at the momentary level (Kerkhof and van Spijker, 2011; Law and Tucker, 2018; Morrison and O’Connor, 2008; Rogers and Joiner, 2017). Moreover, several cross-sectional studies identified rumination as a potential mediating pathway between negative or traumatic life events and SI (Chan et al., 2009; Valderrama et al., 2022; Wang et al., 2020; Yao et al., 2022). However, to our knowledge, no prior research has used a longitudinal framework to examine if rumination indirectly accounts for near-term associations between negative events and SI in daily life.

It is also likely that the extent to which pathways between negative events and rumination are relevant to near-term SI varies considerably across individuals. Although many suicide theories emphasize common pathways of suicide risk (i.e., Joiner, 2005; O’Connor and Kirtley, 2018), research increasingly indicates that suicide risk is a multifinal and highly individualized phenomenon (Kaurin et al., 2022; Millner et al., 2020; Sewall and Wright, 2021). Indeed, recent studies found that common risk pathways for suicide, and the size of observed effects for these risk processes, vary significantly across people (Coppersmith et al., 2024; Kaurin et al., 2022). These findings indicate heterogeneity in risk pathways across individuals. Thus, the degree to which rumination explains the relationship between negative life events and SI intensity likely varies considerably across people. Identifying which individuals are at greater risk of SI after negative life events as a function of rumination could help to inform tailored interventions for individuals most at-risk for suicide. However, to our knowledge, no previous literature has explored person-specific heterogeneity in pathways between negative events, rumination, and SI.

### 1.1. Current study

Taken together, the present study seeks to fill an important gap in prior literature by examining if rumination indirectly accounts for short-term associations between negative events and SI in a high-risk sample of adults with recent psychiatric hospitalization. Using day-level ecological momentary assessment (EMA) data, we hypothesized that (1) negative events would directly associate with daily rumination and daily SI intensity, (2) daily rumination would directly associate with daily SI intensity, and (3) an indirect pathway through rumination would partially account for the daily association between negative events and SI intensity. Given previous research identifying significant heterogeneity in processes that influence short-term risk for suicidality, we integrated group-level (i.e., nomothetic) and person-specific approaches by modeling overall direct and indirect effects across the full sample, and then examining how patterns of effects varied across individual participants.

## 2. Methods

### 2.1. Participants and procedures

Participants were 107 adult psychiatric inpatients who were recruited from a psychiatric hospital in New England as part of a larger study. Study procedures have been detailed elsewhere (Gideon et al., 2019; Wallace et al., 2024). All procedures were approved by the Institutional Review Board of the participating hospital, and informed consent was obtained from all participants. Data from this study can be requested from the National Institute of Mental Health Data Archive (Collection ID #2411). Inclusion criteria were as follows: current admission to a psychiatric inpatient unit; 18–65 years of age; ability to speak, read, and understand English well enough to complete study procedures; considered psychiatrically stable by the inpatient treatment team to participate in research; and comfort with the use of smartphone technology. Exclusion criteria included current manic or psychotic symptoms or cognitive impairments that would preclude informed consent and adequate participation in study procedures. Cognitive impairments and psychotic symptoms were identified through chart review, consultation with participants’ attending physicians, and behavioral observations of participants’ ability to engage with the consent and eligibility procedures. Participants were only invited to enroll in the study if their attending physician provided permission and indicated no concerns about their ability to complete study activities.

Of the 107 participants, 39.25% (n = 42) were admitted to the hospital for suicide attempt, 43.00% (n = 46) were admitted for SI, and 17.75% (n = 19) were admitted for psychiatric reasons not involving suicidality. Prior to hospital discharge, inpatients completed baseline assessments (self-report measures, clinical interviews) and were trained in an EMA protocol using the ilumivu mEMA app, which was installed on either their personal smartphone or a study-provided smartphone. After discharge, participants completed a 65-day EMA protocol, during which they received three randomly timed (i.e., signal contingent) EMA surveys per day across participant-selected waking hours. These thrice-daily EMA surveys assessed self-reports of suicidal thoughts and behaviors, cognitive-affective experiences, and events since the previous assessment. A daily morning check-in survey was also administered via EMA, but the morning surveys assessed other constructs and thus were not included in the current study. While 158 participants enrolled in the study and completed baseline procedures, 51 participants were excluded from the analytic sample due to not providing any EMA data on the study variables. Table 1 presents demographic characteristics for the full baseline and analytic samples, and differences between the samples were minimal.

### 2.2. Measures

#### 2.2.1. Negative events

Negative events were measured via two items in the EMA survey. Participants were first asked the binary item “Since you last completed the questionnaire, have you experienced a negative event or an unpleasant experience?” If participants responded “yes” they were asked, “How negative or unpleasant was the event?” Participants responded on a Likert-style scale ranging from 1 (*very slightly or not at all*) to 5 (*extremely*). Participants were coded 0 if they reported no negative events since the previous survey.

#### 2.2.2. Rumination

Rumination was assessed as a mean score of three EMA items adapted from the Response Styles Questionnaire (Butler and Nolen-Hoeksema, 1994; Nolen-Hoeksema and Morrow, 1991): “I’ve been thinking: ‘about a recent situation, wishing that it had gone better,’ ‘why do I always react this way,’ and ‘why do I have problems other people don’t have’.” Participants responded to each item on a Likert-style scale ranging from 1 (*strongly disagree*) to 5 (*strongly agree*). The rumination items demonstrated good reliability (ω_within_ = 0.75, ω_between_ = 0.89) (Geldhof et al., 2014).

#### 2.2.3. Suicidal ideation intensity

SI intensity was assessed with two EMA items. Participants were first asked the binary item, “Since you last completed a questionnaire, have you wished you were dead or had thoughts about ending your life?” Participants who responded “yes” were then asked, “How intense or powerful were these thoughts?” Participants responded on a Likert-style scale ranging from 1 (*very weak*) to 4 (*strong*). Participants were coded 0 if they reported no SI since the previous survey.

#### 2.2.4. Analytic plan

Data were cleaned using R v4.4.0 (R Core Team, 2024) and analyses were conducted using MPlus v8.10 (Muthén & Muthén, 1998–2017). Prior to analysis, data were evaluated for missingness and statistical assumptions of normality and non-stationarity. To account for sparsity in EMA responses, in which most participants (82%, n = 88 out of 107) responded to an average of one or fewer EMA prompts within a given day, we aggregated each EMA-measured variable to day-level maximums (i.e., the time-lag between observations in models was approximately 24 h) (Glenn et al., 2021; Wallace et al., 2024). Thus, all analytic variables represented self-reports of experiences that occurred within the same day-level window (spanning approximately 12 h).

**Fig. 1.**
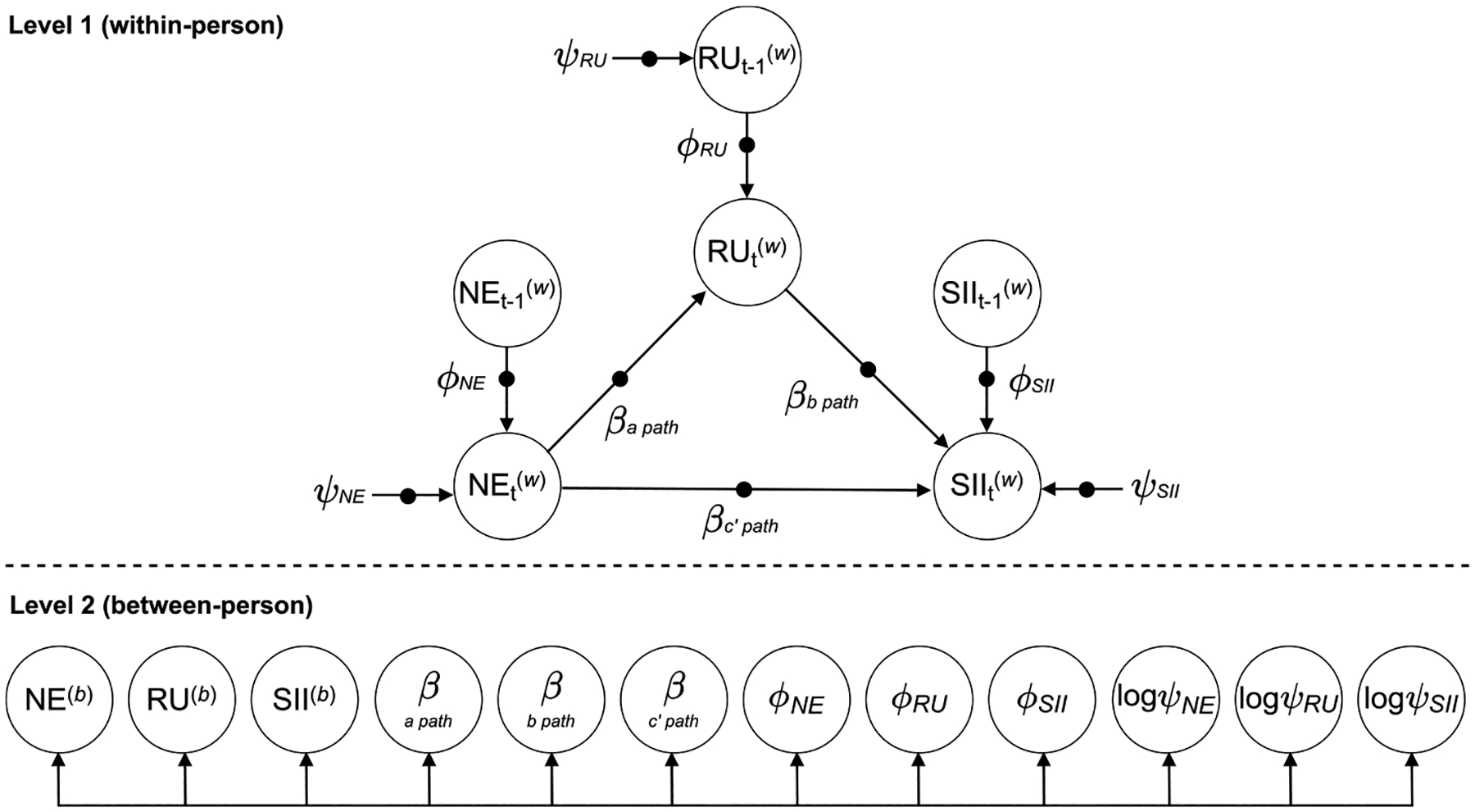
Model overview. We used a two-level Dynamic Structural Equation Modeling framework to estimate the 1-1-1 indirect effects model. The model examined within-person same-day associations between ecologically assessed rumination on negative events (a path), suicidal ideation intensity on rumination (b path), and suicidal ideation intensity on negative events (c’ path). Circles in lines denote random effects, β denotes a bivariate regression slope, ϕ denotes an autoregressive slope, and ψ denotes a residual variance. NE = negative events, RU = rumination, SII = suicidal ideation intensity.

To model hypothesized effects across the full sample, we used a two-level Dynamic Structural Equation Modeling (DSEM) framework to estimate a 1-1-1 indirect effects model of within-person associations between negative events, rumination, and SI intensity (Krull and MacKinnon, 2001; McNeish and Mackinnon, 2022). Fig. 1 presents the conceptual overview for our model. Repeated measures from the day-level EMA variables were nested within-person. At Level 1 (within-person), same-day rumination was regressed onto same-day negative events (a path), same-day SI intensity was regressed onto same-day rumination (b path), and same-day SI intensity was regressed onto same-day negative events (c’ path). Additionally, Level 1 included random variances and random autoregressions (i.e., each variable regressed on itself at the previous timepoint) for all three variables. The random autoregressions were included to account for temporal dependency in the data, since not controlling for spill-over effects from previous timepoints can lead to biased estimates (Asparouhov et al., 2018). At Level 2 (between-person), the three variables (negative events, rumination, SI intensity) and the nine random effects from Level 1 (random regressions, autoregressions, and log residual variances) were allowed to co-vary (see Fig. 1).

We calculated the size of the indirect and total effects in the full model using equations defined by Bolger and Laurenceau (2013). The within-person indirect effect was calculated as the product of the a and b paths plus the covariance of the a and b paths (a*b + cov(ab)). The within-person total effect was calculated as the product of the a and b paths plus the c’ path and the covariance of a and b (a*b + c’ + cov(ab)) (Bolger and Laurenceau, 2013). We then probed how much the direct and indirect effects varied across individual participants by adding a person-specific step to our model. Following recommendations from McNeish and MacKinnon (2022), we used MPlus’ multiple imputation capabilities to calculate and save 500 plausible values for each regression path for each participant to obtain person-specific posterior distributions for the direct and indirect effects (Asparouhov and Muthén, 2010). This approach is conceptually similar to bootstrapping; by using Bayesian estimation to re-estimate model paths 500 times, we were able to calculate each participant’s plausible median effect sizes from their posterior distributions, which we then used to evaluate if the regression paths were null versus present for different people. We visualized the person-specific medians and standard deviations for each effect to explore how much these varied across participants. Post-hoc Wilcoxon tests were then used to evaluate differences in median effect sizes across participants.

Models were estimated using Bayesian estimation with uninformative priors and 20,000 iterations with 50% burn-in (McNeish and Hamaker, 2020). Latent person mean centering was used for all variables, and model convergence was evaluated by ensuring Potential Scale Reduction values were <1.1 (Hamaker et al., 2018; McNeish and Mackinnon, 2022). As noted above, the three EMA variables were aggregated to the day-level. Modeling time-lags in the Level 1 bivariate regressions, as is typical in longitudinal mediation (McNeish and Mackinnon, 2022), would represent effects occurring across approximately 24–48 h. However, prior research indicates that rumination and negative events can influence SI on shorter timescales (e.g., hours or same day) (Franz et al., 2021; Rogers et al., 2022). There is need for research on *short*-*term* risk for suicidal thoughts and behaviors to improve timely risk detection and intervention (Kleiman and Nock, 2018). Given this, we elected to not lag the Level 1 bivariate regressions, and these represent effects occurring *within the same day*. We do not interpret temporal directionality (Cain et al., 2017), and results provide information about same-day indirect associations between variables while allowing slopes to vary across participants and adjusting for random autoregressions and variances.

## 3. Results

### 3.1. Descriptive statistics

Table 2 presents descriptive statistics, intraclass correlations, and bivariate correlations for the study variables. In total, the model included 1961 observations across the 107 participants. The average cluster size was 18.33 (*SD* = 16.83, median = 11, minimum = 1, maximum = 65), indicating that, on average, participants provided data on 18.33 out of 65 days during the EMA protocol. Additional details on EMA compliance in the sample are described elsewhere (Wallace et al., 2024). Nearly three quarters of participants, 74.44% (n = 80), reported experiencing a negative event in the EMA protocol, with 58.88% (n = 63) reporting a negative event on more than one day. Approximately half the sample, 49.53% (n = 53), reported SI in the EMA protocol, and 32.71 % (n = 32) reported SI on more than one day. While the negative events and SI intensity variables were both overdispersed (see Table 2), Bayesian estimation does not rely on assumptions of normality (Muthén, 2010). Non-stationarity was not indicated (i.e., significant time trends were not observed), so variables were not detrended prior to analysis. Missing data were handled using Bayesian estimation with uninformative priors, which uses all available observations for model estimates (McNeish and Hamaker, 2020).

### 3.2. Model results

Standardized and unstandardized estimates for the 1-1-1 indirect effects model in the full sample are shown in Table 3. Both the fixed and random autoregressions and variabilities (log residual variances) were significant for negative events, rumination, and SI intensity. Thus, previous observations influenced subsequent observations, and participants differed in the strength of these autoregressive effects and how much their reports of each construct varied across time (see Table 3). All three bivariate regression paths were significant (i.e., the 95% Bayesian credibility interval [CI] excluded 0) and in the hypothesized direction. Negative events were associated with higher same-day rumination (a path; standardized fixed effect = 1.174, 95% CI = 0.786–1.622), higher rumination was associated with higher same-day SI intensity (b path; standardized fixed effect = 0.865, 95% CI = 0.620–1.109), and negative events were associated with higher same-day SI intensity (c’ path; standardized fixed effect = 0.802, 95% CI = 0.457–1.090). The random effects were significant for all three bivariate regressions, indicating heterogeneity in these effects across the sample. In the full model, the indirect effect of rumination (indirect effect unstandardized estimate = 0.026) was significant and represented 38.058% of the total effect (total effect unstandardized estimate = 0.068). Thus, a substantial proportion of the association between negative events and SI appeared to be accounted for by an indirect pathway through rumination.

**Fig. 2.**
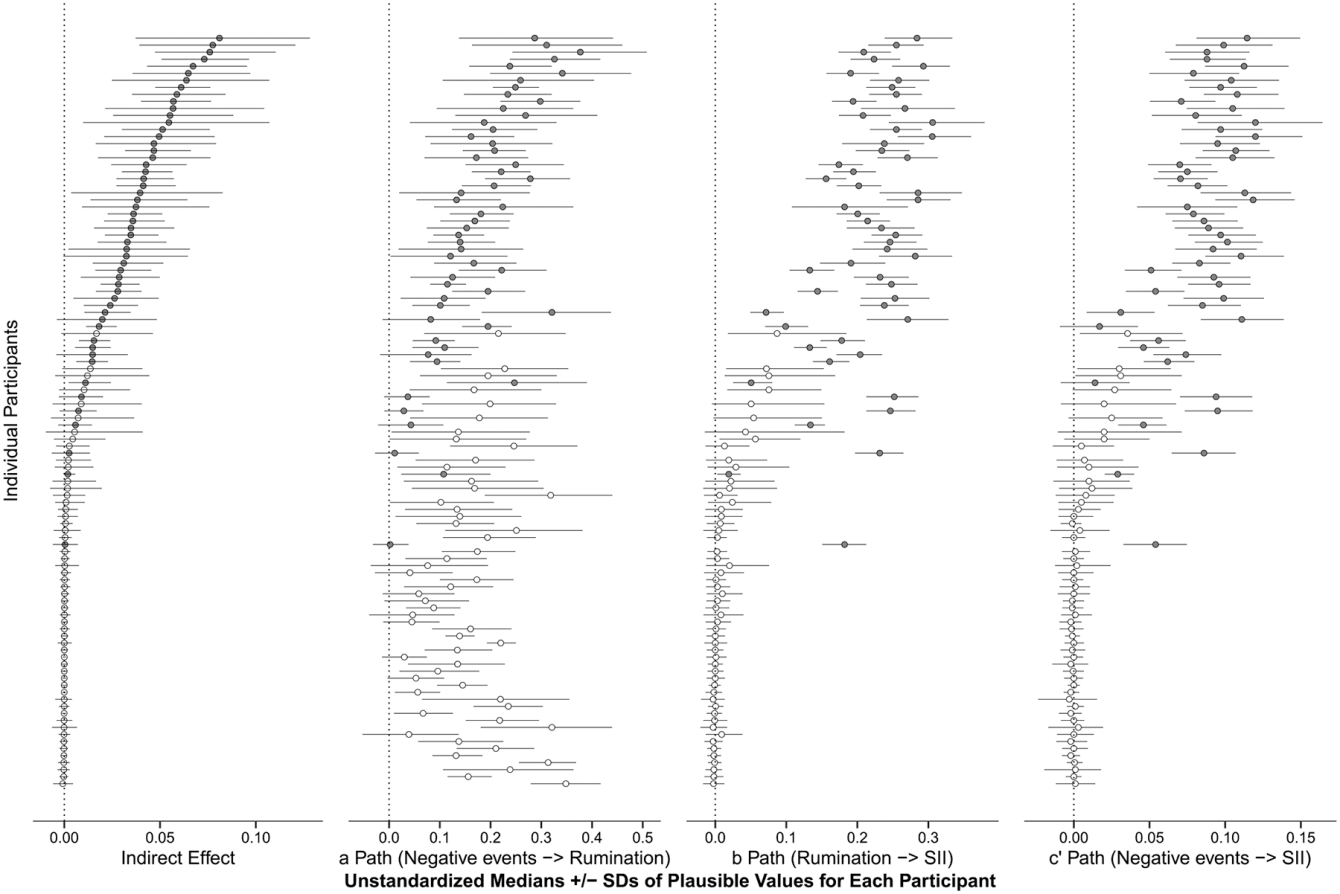
Person-specific medians and standard deviations of plausible values for the direct and indirect effects for the 1-1-1 indirect effects model (shown in Fig. 1) across the *N*=107 participants. Each circle represents the median unstandardized effect for an individual participant, calculated across 500 plausible values obtained using multiple imputation capabilities with Bayesian estimation in Mplus (Asparouhov and Muthén, 2010; McNeish and Mackinnon, 2022). Error bars represent standard deviations (SDs). Gray-filled circles represent participants who reported any SI in the EMA surveys, while empty circles represent participants who reported no SI in the EMA. SII = suicidal ideation intensity.

To visualize how much the hypothesized effects varied across people in the sample, Fig. 2 presents person-specific medians and standard deviations for plausible values of the direct and indirect effects for each individual participant (McNeish and Mackinnon, 2022). Participants varied considerably in their relative medians for the three direct effects (a path, b path, and c’ path) and the indirect effect. Nearly all participants, n = 91 or 85%, had a positive association (median ± SD > 0) between same-day negative events and rumination (a path), suggesting that rumination in the context of negative events is salient for most inpatients in the post-discharge period. However, only a portion of the sample demonstrated positive (median ± SD > 0) associations for the indirect effect (n = 44 or 41%) or direct effects between rumination and SI intensity (b path; n = 58 or 54%), and between negative events and SI intensity (c’ path; n = 54 or 50.5%).

Recall that nearly half the sample (n = 53) reported one or more instances of SI in the EMA protocol and, given that SI intensity was our primary outcome, the presence or absence of SI during the study period could have influenced individual participants’ results. Thus, we further probed heterogeneity in the sample by visualizing how the person-specific estimates varied based on whether participants reported any SI within the EMA protocol (see Fig. 2). These person-specific results indicated that the indirect effect was present (i.e., median indirect effect ± SD > 0) for most participants who reported SI in their EMA responses, and was not present (i.e., null; median indirect effect ± SD included 0) for most participants who denied SI throughout their EMA surveys. In other words, nearly all participants who reported SI during the study (*n* = 44 out of 53) evidenced an indirect effect, in which rumination accounted for part of the association between daily negative events and SI intensity. Unsurprisingly, among participants who endorsed SI, the indirect effect of rumination was larger for those who reported stronger bivariate associations between negative events, rumination, and SI intensity. Post-hoc Wilcoxon tests indicated that, compared to participants who denied SI in their EMA responses, participants who reported SI had larger median effect sizes for the b path (W = 24.0, *p* < .001, SI group median = 0.23, no-SI group median = 0.00), c’ path (W = 20.5, *p* < .001, SI group median = 0.09, no-SI group median = 0), and indirect effect (W = 62.0, *p* < .001, SI group median = 0.04, no-SI group median = 0.00). The a path did not significantly differ between participants who did versus did not endorse SI (W = 1183.5, *p* = .12, SI group median = 0.18, no-SI group median = 0.14).

## 4. Discussion

This study tested if rumination indirectly accounts for within-person (i.e., intraindividual) associations between negative events and SI at the daily level. Our initial hypotheses were supported in the full sample model, in which negative events had direct associations with same-day rumination and SI intensity, rumination had a direct association with same-day SI intensity, and the indirect pathway through rumination accounted for a large percent (38%) of the total effect between negative events and SI intensity. Person-specific results indicated that the magnitude of these effects varied substantially across individual participants, highlighting the importance of examining heterogeneity across people in short-term suicide risk models. Nonetheless, most participants who reported any SI during the study period evidenced an indirect effect, indicating rumination is a salient modifiable factor that increases risk for SI in the context of negative life events for a subset of individuals.

Although negative life events are an established risk factor for SI (Liu and Miller, 2014), our results may explain why only some individuals experience SI after negative or stressful experiences (Howarth et al., 2020). Results from our full sample model indicate that, in general, participants who reported greater tendency to engage in rumination (i. e., repetitive negative thinking) had a greater risk of reporting higher SI intensity when they had a more severe negative event occur on the same day. Thus, for a subset of individuals, experiencing SI during or after a negative event may partly be a function of intently focusing on and struggling to disengage from repetitive thoughts about the upsetting experience. These findings from our full sample model are consistent with the emotional cascade model of suicide risk (Selby and Joiner, 2013), in which the way that some individuals *engage with* their thoughts about negative events may perpetuate or exacerbate their distress and concomitant SI.

Nevertheless, while rumination accounted for part of the relationship between negative events and SI in the full sample model, the indirect effect of rumination did not *completely* account for the total effect of negative events on SI, suggesting other factors also influence these processes. This idea is also supported by our person-specific analyses, which revealed that most participants (85%) in the sample reported higher rumination in direct relation to negative events, but only 50.5% of the sample demonstrated a direct association between negative events and SI intensity. Thus, while results indicate that psychiatric inpatients are generally prone to rumination on the same day as unpleasant events during the weeks after hospital discharge, the presence of rumination alone does not fully explain why some participants experienced SI. Contextual risk factors, such as the type of negative event, co-occurrence of multiple life stressors, or presence of psychiatric symptoms, could importantly impact individuals’ cognitive and emotional experiences (Kleiman et al., 2017; von Klipstein et al., 2024). Further, contextual protective factors such as using a coping skill or receiving social support (Kleiman et al., 2014) after a negative event may protect against concurrent or subsequent SI. These contextual factors should be considered in future models of negative events, rumination, and SI.

Nonetheless, while some participants experienced rumination on days with negative events *without* having SI, nearly all participants who reported SI during post-hospitalization study period had an observable indirect effect of rumination. Thus, our person-specific analyses reinforce that rumination plays a role in the association between negative events and SI. It is also noteworthy that the size of the direct and indirect effects varied substantially across participants, showing that the degree to which rumination increases risk for SI in the context of negative events varies across people. This heterogeneity across participants would have been obscured by only using group-level (i.e., nomothetic) techniques to model these processes. Our results corroborate that risk for SI is a highly individualized phenomenon, and personalized risk models are thus likely to outperform group-level approaches for meaningfully detecting short-term risk (Sewall and Wright, 2021). Thus, findings provide support for applying person-specific analytic approaches to intensive longitudinal data to understand nuanced heterogeneity in short-term suicide risk processes (Coppersmith et al., 2024; Sewall and Wright, 2021).

Results have implications for clinically monitoring and intervening on risk for suicide in psychiatric inpatients after hospitalization. While negative life events and stressors cannot necessarily be avoided, rumination is a modifiable cognitive process that can be reduced through existing clinical interventions. However, given that the degree to which rumination is associated with SI varies across individuals, understanding how these constructs are related for a particular individual suggests the importance of personalized intervention. For example, behavioral chain analysis, one of the mechanisms of change in dialectical behavior therapy, may permit individuals a deeper understanding of the factors impacting their rumination (Mattei and Sposato, 2020). Further, individuals can learn to recognize and reduce rumination through evidence-based skills such as cognitive restructuring, acceptance of thoughts, and distress tolerance (Bryan and Rudd, 2018; Linehan, 2015). Helping patients reduce ruminative thinking could thus provide a protective effect against SI in the context of negative events (Rogers et al., 2022). This is especially relevant for our sample of psychiatric inpatients, as the period after psychiatric hospitalization is often characterized by significant life stressors (Nordentoft et al., 2021) and elevated risk for suicide (Chung et al., 2017; Olfson et al., 2016).

### 4.1. Limitations and future directions

Our results should be interpreted in the context of some limitations. First, our sample largely consisted of participants with social identities that represent the dominant culture and/or are overrepresented in mental health literature (81% White, 69% heterosexual, 65% female assigned at birth; see Table 1), and gender identity was not available in the data. Individuals with non-dominant and intersecting non-dominant social identities have elevated risk for suicide (Centers for Disease Control and Prevention, 2024). Replicating our findings in more diverse samples will be important for inferring generalizability of these results. Additionally, we used EMA data aggregated to the day-level and examined same-day direct and indirect effects, which precluded interpreting temporal ordering and directionality of the observed effects. It is also possible that the effect sizes for these risk processes are different across shorter (e.g., minutes or hours) or longer (e.g., weekly) timescales. Future research that employs time-lagged models, and investigates the specific timescales at which these effects are largest (e.g., Langener et al., 2024), would provide valuable information about how and when negative events increase risk for rumination and SI in daily life.

Analyses also did not control for covariates that might influence associations between negative events, rumination, and SI. For example, we measured negative events using brief EMA items that did not consider the type or long-term impacts of these negative experiences; daily unpleasant experiences may impact suicide risk differently than major life events, such as those measured by the Life Events Checklist (e. g., loss of loved one, major illness) (Gray et al., 2004). Further, while rumination and suicidality are transdiagnostic phenomena (Caudle et al., 2024), psychiatric diagnoses and symptoms could have impacted participants’ experiences of the study variables, and future research would profit from including psychiatric symptoms in suicide risk models. Future studies that consider additional contextual factors in analyses (e.g., social support, psychiatric symptoms, type of negative event experienced) would also help to inform the development of personalized risk models and personally-tailored interventions for suicide after negative life events (Coppersmith et al., 2022). Finally, we used brief self-report measures for all study variables. Self-report measures can suffer from recall bias, and the use of single-item variables for negative events and SI intensity prevented examining reliability of these constructs (Dejonckheere et al., 2022).

## 5. Conclusions

To our knowledge, the current study is the first to examine day-level indirect pathways between negative events, rumination, and SI intensity in the period following psychiatric hospitalization. Results suggest that the indirect pathway through rumination accounts for a large percent of the direct pathway between negative events and SI intensity. However, the strength of these effects varied across individual participants and were larger among participants who endorsed SI during the study period, pointing to the importance of using person-specific analytic approaches with intensive longitudinal datasets. Personalized interventions focused on reducing ruminative thinking in the context of negative life events may buffer risk for SI among individuals who are at elevated risk for suicide. Future research should evaluate these pathways across different timescales (e.g., within hours of a negative event) with the goal of administering interventions for suicide when they are most needed.

## Figures and Tables

**Table 1:** Demographic and clinical characteristics of the baseline and analytic samples.

Baseline Characteristic	Baseline Sample (*N* = 158)	Analytic Sample (*N* = 107)
	*M* (*SD*) or *n* (%)	*M* (*SD*) or *n* (%)
Age (in years)	35.52 (14.50)	35.85 (14.44)
*n* missing	0	0
Sex assigned at birth^1^		
Female	101 (64 %)	70 (65 %)
Male	54 (34 %)	35 (33 %)
Other	3 (2 %)	2 (2 %)
*n* missing	0	0
Sexual orientation		
Sexual minority	45 (30 %)	33 (31 %)
Straight or heterosexual	103 (70 %)	72 (69 %)
*n* missing	10	2
Race		
American Indian or Alaska	3 (2 %)	3 (3 %)
Native		
Asian	4 (2 %)	2 (2 %)
Black or African American	5 (3 %)	3 (3 %)
Multiracial/Other	15 (10 %)	12 (11 %)
Native Hawaiian or Other Pacific Islander	0 (0 %)	0 (0 %)
White	130 (83 %)	87 (81 %)
*n* missing	1	0
Ethnicity		
Hispanic or Latino	14 (9 %)	11 (10 %)
Not Hispanic or Latino	133 (84 %)	93 (87 %)
Unknown or not reported	11 (7 %)	3 (3 %)
*n* missing	0	
Education (total # of years in school)	13.78 (2.91)	13.96 (3.21)
*n* missing	12	4
Previous suicide attempt		
No	95 (61 %)	62 (58 %)
Yes	62 (39 %)	44 (42 %)
*n* missing	1	1

*Note*: The analytic sample excluded 51 participants who completed baseline procedures but provided no valid ecological momentary assessment data. All sample characteristics were measured at baseline.

1 Gender identity was not available.

**Table 2:** Descriptive statistics, bivariate correlations, and intraclass correlations for study variables.

	*M* (*SD*)	*Multilevel correlations and ICCs*		
		Negative events	Rumination	Suicidal ideation intensity
Negative events	0.719 (1.711)	0.324	0.263*	0.398*
Rumination	1.254 (1.120)	0.303*	0.613	0.352*
Suicidal ideation intensity	0.278 (0.828)	0.257*	0.246*	0.453

*Note*: N_between_ = 107 and N_within_ = 1961. All variables were measured via ecological momentary assessment and aggregated to the day-level. Within-person correlations are shown in the lower triangle, between-person correlations are shown in the upper triangle, and intraclass correlations (ICCs) are shown in the diagonal. Multilevel correlations and ICCs were estimated in MPlus.

* All correlation coefficients had *p* < 0.05.

**Table 3:** Estimates for 1-1-1 indirect effects model examining within-person pathways between negative events, rumination, and suicidal ideation (N_between_ = 107, N_within_ = 1961).

	Unstandardized estimate (95 % CI)	Standardized estimate (95 % CI)
Means (Fixed effects)		
Negative events_t_ → Rumination_t_ (β a path)	**0.170** (0.113, 0.235)	**1.174** (0.786, 1.622)
Rumination_t_ → SII_t_ (β b path)	**0.114** (0.078, 0.155)	**0.865** (0.620, 1.109)
Negative events_t_ → SII_t_ (β c’ path)	**0.043** (0.028, 0.071)	**0.802** (0.457, 1.090)
Negative events_t-1_ → Negative events_t_ (ϕ_*NE*_)	**0.192** (0.094, 0.286)	**0.567** (0.252, 0.920)
Rumination_t-1_ → Rumination_t_ (ϕ_*RU*_)	**0.371** (0.263, 0.473)	**1.030** (0.622, 1.536)
SII_t-1_→ SII_t_ (ϕ_*SII*_)	**0.123** (0.044, 0.200)	**0.435** (0.152, 0.718)
Negative events variability (logψM_*NE*_)	**−0.638** (−1.252, −0.030)	**−0.208** (−0.408, −0.009)
Rumination variability (logψ_*RU*_)	**−1.390** (−1.59, −1.191)	**−1.609** (−2.037, −1.222)
SII variability (logψ_*SII*_)	**−3.166** (−3.833, −2.492)	**−0.942** (−1.195, −0.696)
Negative events mean (μ_*NE*_)	**0.496** (0.382, 0.632)	**1.233** (0.890, 1.623)
Rumination mean (μ_*RU*_)	**2.019** (1.816, 2.225)	**2.155** (1.740, 2.592)
SII mean (μ_*SII*_)	**0.134** (0.098, 0.184)	**0.871** (0.628, 1.110)
Variances (Random effects)		–
Negative events_t_ → Rumination_t_ (β a path)	**0.021** (0.010, 0.047)	–
Rumination_t_ → SII_t_ (β b path)	**0.018** (0.009, 0.032)	–
Negative events_t_ → SII_t_ (β c’ path)	**0.003** (0.002, 0.012)	–
Negative events_t-1_ → Negative events_t_ (ϕ_*NE*_)	**0.115** (0.072, 0.188)	–
Rumination_t-1_ → Rumination_t_ (ϕ_*RU*_)	**0.130** (0.075, 0.222)	–
SII_t-1_→ SII_t_ (ϕ_*SII*_)	**0.081** (0.052, 0.127)	–
Negative events variability (logψ_*NE*_)	**9.471** (6.876, 13.366)	–
Rumination variability (logψ_*RU*_)	**0.751** (0.493, 1.176)	–
SII variability (logψ_*SII*_)	**11.292** (8.356, 15.662)	–
Negative events mean (μ_*NE*_)	**0.165** (0.078, 0.311)	–
Rumination mean (μ_*RU*_)	**0.876** (0.618, 1.271)	–
SII mean (μ_*SII*_)	**0.024** (0.015, 0.046)	–

*Note:* See Fig. 1 for a visual overview of the model. Bolded values represent effects interpreted as significant (i.e., the CI excludes 0). The three bivariate regression paths represent contemporaneous (same-day) effects, while autoregressions represent lagged (across-day) effects. Unstandardized estimates reflect the original scales for each variable, while standardized estimates reflect the number of between-level standard deviations the average of each estimate differs from zero (Hamaker et al., 2023). CI = Bayesian credibility interval, SII = suicidal ideation intensity, β denotes a bivariate slope, ϕ denotes an autoregressive slope, ψ denotes a residual variance, and μ denotes an overall mean.

## Data Availability

The data that support the findings of this study are available from the senior author, HTS, upon reasonable request, and can be requested through the NIMH Data Archive (Collection #2411).
